# Natural infections with different *Plasmodium* species induce antibodies reactive to a chimeric *Plasmodium vivax* recombinant protein

**DOI:** 10.1186/s12936-021-03626-0

**Published:** 2021-02-12

**Authors:** Jessica N. McCaffery, Balwan Singh, Douglas Nace, Alberto Moreno, Venkatachalam Udhayakumar, Eric Rogier

**Affiliations:** 1 Emory Vaccine Center, Yerkes National Primate Research Center, Emory University, 954 Gatewood Road, Atlanta, GA 30329 USA; 2 Malaria Branch, Division of Parasitic Diseases and Malaria, Centers for Disease Control and Prevention, Atlanta, GA 30329 USA; 3 Division of Infectious Diseases, Department of Medicine, Emory University, 69 Jesse Hill, Jr. Drive, Atlanta, SEGA 30303 USA

**Keywords:** Malaria, *Plasmodium vivax*, Chimeric protein, Serology, Multiplex, Seroepidemiology

## Abstract

**Background:**

As malaria incidence and transmission in a region decreases, it becomes increasingly difficult to identify areas of active transmission. Improved methods for identifying and monitoring foci of active malaria transmission are needed in areas of low parasite prevalence in order to achieve malaria elimination. Serological assays can provide population-level infection history to inform elimination campaigns.

**Methods:**

A bead-based multiplex antibody detection assay was used to evaluate a chimeric *Plasmodium vivax* MSP1 protein (PvRMC-MSP1), designed to be broadly immunogenic for use in vaccine studies, to act as a pan-malaria serological tool based on its ability to capture IgG in plasma samples obtained from naturally exposed individuals. Samples from 236 US travellers with PCR confirmed infection status from all four major *Plasmodium* species infecting humans, *Plasmodium falciparum* (n = 181)*, Plasmodium vivax* (n = 38), *Plasmodium malariae* (n = 4), and *Plasmodium ovale* (n = 13) were tested for IgG capture using PvRMC-MSP1 as well as the four recombinant MSP1-19 kD isoforms representative of these *Plasmodium* species.

**Results:**

Regardless of infecting *Plasmodium* species, a large proportion of plasma samples from infected US travellers provided a high assay signal to the PvRMC-MSP1 chimeric protein, with 115 high responders out of 236 samples assessed (48.7%). When grouped by active infection, 38.7% *P. falciparum-*, 92.1% of *P. vivax-*, 75.0% *P. malariae-*, and 53.4% of *P. ovale-*infected individuals displayed high assay signals in response to PvRMC-MSP1. It was also determined that plasma from *P. vivax*-infected individuals produced increased assay signals in response to the PvRMC-MSP1 chimera as compared to the recombinant PvMSP1 for 89.5% (34 out of 38) of individuals. PvRMC-MSP1 also showed improved ability to capture IgG antibodies from *P. falciparum*-infected individuals when compared to the capture by recombinant PvMSP1, with high assay signals observed for 38.7% of *P. falciparum-*infected travellers in response to PvRMC-MSP1 IgG capture compared to just 1.1% who were high responders to capture by the recombinant PvMSP1 protein.

**Conclusions:**

These results support further study of designed antigens as an approach for increasing sensitivity or broadening binding capacity to improve existing serological tools for determining population-level exposure to *Plasmodium* species. Including both broad-reacting and *Plasmodium* species-specific antigen-coated beads in an assay panel could provide a nuanced view of population-level exposure histories, an extensive IgG profile, and detailed seroestimates. A more sensitive serological tool for detection of *P. vivax* exposure would aid malaria elimination campaigns in co-endemic areas and regions where *P. vivax* is the dominant parasite.

## Background

Malaria morbidity and mortality remain a major public health problem globally. According to the World Health Organization (WHO) estimates, there were 229 million cases of malaria in 2019, which resulted in 409,000 deaths, primarily in children under five years of age [1].

Despite the high death toll caused by malaria, 19 countries attained 3 consecutive years without indigenous cases of malaria, with China and El Salvador the latest to countries to achieve malaria elimination and becoming certified as malaria free by the WHO in 2019 [1]. Unfortunately, as malaria incidence and transmission in a region decreases, it becomes increasingly difficult to identify areas of active transmission due to the rise in asymptomatic infections and lower numbers of individuals seeking treatment for malarial illness [2–9]. Improved methods for identifying and monitoring foci of active malaria transmission are particularly needed in these areas of low parasite prevalence.

Entomological inoculation rates (EIR), which measure the mean number of infectious mosquito bites per individual over time, remain a widely-accepted measure of transmission, but lack precision due to heterogeneous mosquito distributions [10]. Furthermore, this method is not without practical and ethical considerations, as it requires mosquito trapping using adult volunteers. Additionally, EIR can be difficult to extrapolate to paediatric populations [11, 12]. Co-endemic patterns of different *Plasmodium* species also pose added difficulties for malaria control efforts as vector-based interventions targeted toward *Plasmodium falciparum* are less efficacious against *Plasmodium vivax* [13]. In sub-Saharan Africa, *P. falciparum* is by far the most prevalent malaria parasite, but *P. vivax*, *Plasmodium malariae,* and *Plasmodium ovale* can also be co-endemic [14]. Human populations in many other parts of the world are also subjected to the burden of multiple *Plasmodium* species, complicating epidemiological studies and elimination interventions as indicators only exist for the predominant parasite species [8, 15, 16].

There has been an increase in the use of serological and antibody-based detection assays for measuring exposure and determining transmission intensity in regions of malaria control implementation and those undergoing elimination campaigns [17–19]. Unlike diagnostic assays, which look for the presence or absence of active infection, these quantitative immunoassays offer several advantages for epidemiological studies, including more robust data generation and allowing for estimation of the individual- and population-level malaria exposure history [8, 20–23]. The ability to use dried blood spot samples also makes serological methods pragmatic for field sample collection and laboratory processing [24]. One or multiple recombinant proteins are typically used to detect anti-*Plasmodium* antibodies in sera or blood samples, and common species-specific targets include circumsporozoite protein (CSP), apical membrane antigen 1 (AMA1), and merozoite surface proteins (MSP1, MSP2, and MSP3) [17, 18, 21, 25, 26]. IgG responses to the recombinant MSP1 19 kD (MSP1_19_) isoforms for the four human *Plasmodium* species (*P. falciparum, P. vivax, P. malariae,* and *P. ovale*) have been found to be largely species-specific, giving more confidence in population seroestimates even when these four *Plasmodium spp.* are co-endemic [26].

However, unlike malaria rapid diagnostic tests, which have begun to assess pan-malaria antigen levels [27], serological studies have yet to include pan-malaria (pan-*Plasmodium*) antigens into existing antigen panels for antibody capture. The addition of pan-malaria antigens for IgG detection could improve malaria exposure estimates to aid in malaria elimination campaigns, which aim to eliminate all malaria transmission within a region, not just an individual species. Recently, the use of designed chimeric *Plasmodium* proteins as serosurveillance tools has been explored, including a multi-epitope chimeric antigen that contained epitopes from eight *P. falciparum* antigens [28, 29]. While this antigen was well-recognized by *P. falciparum*-infected individuals in endemic regions along the China-Myanmar border, it displayed limited cross-reactivity with *P. vivax*-infected individuals [28].

This study aims to assess the ability of a novel chimeric antigen based on *P. vivax* MSP1 to serve as a pan-malaria surveillance tool that can act as a marker for exposure to any *Plasmodium* species infecting humans. A chimeric *P. vivax* MSP1 protein (designated PvRMC-MSP1) has been previously designed, cloned, and expressed for the primary purposes of malaria vaccine studies. This chimeric protein contains an extended version of PvMSP1_19_ containing two T helper epitopes present in the PvMSP1_33_ fragment of the native protein, five promiscuous T cell epitopes (capable of binding multiple MHC Class II alleles) arrayed in tandem, and a C-terminal (NANP)_6_ affinity tag derived from the central repeat region of the *P. falciparum* CSP [30]. The T cell epitopes in PvRMC-MSP1 represent different regions of PvMSP1 able to bind to several MHC class II alleles, with two epitopes also functioning as B cell epitopes [31]. A previous report showed that PvRMC-MSP1 was recognized by antibodies from individuals living in malaria-endemic areas of Brazil with no indication of genetic restriction based on HLA-DRB1 and HLA-DQB1* allele frequencies [30]. Due to the evidence that PvRMC-MSP1 can capture IgG from naturally exposed individuals, this study evaluates the ability of PvRMC-MSP1 to induce antibodies that bind to non-vivax-specific antigenic targets and the ability of PvRMC-MSP1 to capture naturally acquired IgG antibodies from exposed individuals with known infection status from all four human *Plasmodium* species. This qualitative study aims to demonstrate the potential of engineered antigens for use in serological surveys.

## Methods

### Ethics statements

All animal protocols that include experimental animal procedures using mice and rabbits were carried out in accordance with the US Animal Welfare Act and approved by the Institutional Animal Care and Use Committee of Emory University, protocol YER-2002483-092616GN, and followed accordingly.

Samples with or without PCR-confirmed malaria infection were described previously [32], and determined by the CDC Human Subjects office to be research that does not involve human subjects (2017–192). No contact information nor access to personal identifiers were available to laboratory staff from the Centers for Disease Control and Prevention or Emory University. All 236 plasma samples from patients with PCR confirmed infection were obtained from symptomatic individuals upon presenting to a health care provider during the acute phase infection. Anonymous sera samples (N = 92) from US blood donors with no history of foreign travel were used to define a seronegative malaria-naïve population. A *P. falciparum* hyperimmune sera pool was created using sera samples collected from adults living in *P. falciparum* malaria-endemic regions around the world.

### Structure of *P. vivax *recombinant chimera and other *Plasmodium* antigens used in the study

**Fig. 1:**
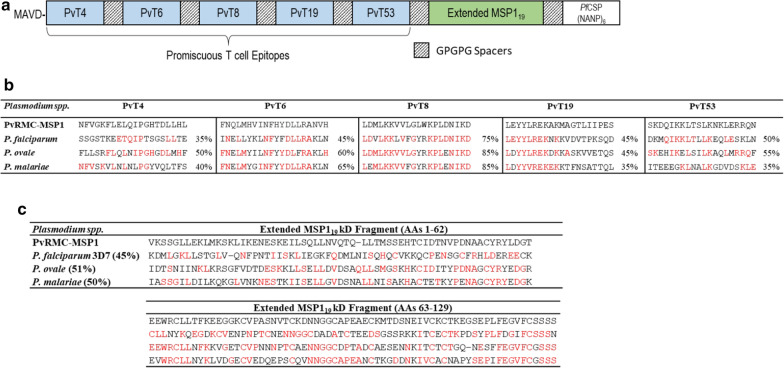
Schematic of the PvRMC-MSP1 chimeric protein and alignments of orthologous sequences**. a** The entire length of the chimera shown with T cell epitopes represented as blue boxes with the *P.* vivax T cell epitope designation (PvT) listed inside each box. **b** Alignment of PvRMC-MSP1 T cell epitopes with orthologous regions from other human *Plasmodium* species. **c** Alignment of 19 kD fragment within PvRMC-MSP1 with MSP1_19_ regions in other human *Plasmodium* species and the partial 33 kD segment

The design of PvRMC-MSP1 has been previously described, based on the *P. vivax* Belem sequence (GenBank: XP_001614842.1), for use as a malaria vaccine candidate [30] (Fig. 1). PvRMC-MSP1 includes the following segments: (1) Methionine and alanine were included as the first two amino acids to provide both the start signal and decrease degradation during synthesis in *Escherichia coli*, and valine and aspartic acid were introduced downstream as part of the cloning strategy; (2) Five promiscuous T cell epitopes [30, 31], designated PvT4 (N_78_-L_97_), PvT6 (F_118_-H_137_), PvT8 (L_158_-D_177_), PvT19 (L_378_-S_397_), and PvT53 (S_1058_-N_1077_) linked in tandem and separated by GPGPG spacers; 3) An extended version of *P. vivax* MSP1_19_ fragment including two T helper epitopes derived from the MSP1_33_ fragment; 4) The peptide (NANP)_6_, representing the major repeat domain derived from the *P. falciparum* circumsporozoite protein was included at the C-terminal end as an additional affinity purification tag. In addition to GPGPG spacers included between the promiscuous T cell epitopes, GPGPG spacers were used to separate all sequences described to enhance the stability of the protein, preserve epitope conformation, and assist with antigen processing. The PfMSP1, PvMSP1, PmMSP1, PoMSP1, and PfCSP antigens have all been used in previous studies and were produced as described before [26, 33].

### Antigen coupling to magnetic beads

All antigens were covalently linked to MagPlex (magnetic) microspheres (Luminex Corp., Austin, TX) as described previously [34]. Magnetic beads were pulse vortexed before being transferred to a microcentrifuge tube and centrifuged for 90 s at 13,000 × *g*. The supernatant was removed and beads were washed with 0.1 M sodium phosphate, pH 6.2 (NaPO_4_). Beads were activated by suspension in NaPO_4_ with 5 mg/ml of EDC (1-ethyl-3-[3-dimethylaminopropyl] carbodiimide hydrochloride) and 5 mg/mL sulfo-NHS (sulfo N-hydroxylsulfosuccinimide) and incubating with rotation for 20 min at room temperature (RT) protected from light. After wash with 50 mM 2-(N-morpholino)ethanesulfonic acid (MES), 0.85% NaCl, pH 5.0, beads were suspended in this same buffer to a final volume of 1 ml, and the appropriate amount of each antigen added: PvRMC-MSP1 (20 µg), PfMSP1 (20 µg), PvMSP1 (20 µg), PmMSP1 (20 µg), PoMSP1 (20 µg), PfCSP (30 µg). Unlike the recombinant MSP1 proteins, for PfCSP, a synthetic peptide containing six copies of the major repeat region, (NANP)_6_, was used. For the PfCSP (NANP)_6_ antigen, a higher amount was determined to be required for the bead coupling reactions so that sufficiently high assay signals could be detected. Beads were rotated for 2 h at RT protected from light, then washed and suspended in PBS with 1% bovine serum albumin (BSA) and incubated for 30 min at RT by rotation. Beads were washed with storage buffer (PBS, 1% BSA, 0.02% sodium azide and 0.05% Tween-20) and suspended in storage buffer containing protease inhibitors (200 µg/mL Pefabloc, 200 µg/ml EDTA, 1 µg/mL pepstatin A and 1 µg/mL leupeptin) and stored at 4 °C.

### Mouse and rabbit immunizations

The immunogenicity of the PvRMC-MSP1 has been previously reported in mice [30]. Female BALB/c (H-2^d^) mice aged 6–8 weeks were obtained from Charles River (Wilmington, MA). Mice were immunized at days 0, 20, and 40, with 20 μg of PvRMC-MSP1 emulsified with the adjuvant Montanide ISA 51 VG at a 1:1 volume ratio. All immunizations were administered subcutaneously at the base of the tail and in the interscapular area. Sera were collected prior to the first immunization and 20 days after the final immunization. Sera from pre-immune timepoints were used as negative controls for multiplex assays.

Rabbits were immunized four times with PvRMC-MSP1 at 20-day intervals. Sera were obtained prior to the first immunization and after the final immunization. All rabbit immunizations and sera collection were carried out by Covance (Princeton, NJ). Rabbit polyclonal IgG antibodies were purified from sera collected after the final immunization by protein A affinity chromatography using the Affi-Gel Protein A MAPS II kit (BioRad) according to the manufacturer’s instructions. The total IgG solution obtained from the elution was then dialyzed in PBS.

### Multiplex antibody detection assays

The standard Multiplex Binding Assay was performed as described previously in flat bottom BioPlex Pro 96 well plates (Bio-Rad, Hercules, CA) [34]. All human samples were assayed at a standard 1:400 dilution of plasma in blocking buffer (Buffer B: PBS, 0.05% Tween 20, 0.5% polyvinylpyrrolidine, 0.5% poly(vinyl) alcohol, 0.1% casein, 0.5% BSA, 0.02% NaN_3_, and 3 µg/mL *E. coli* extract to prevent nonspecific binding). Wash steps included attaching the assay plate to a handheld magnet (Luminex Corp, Austin, TX), the addition of 100 µl wash buffer PBST (PBS 1X, 0.05% Tween-20) to each well, then gentle tapping on the side of the plate to facilitate magnetization of beads, and inverting the plate to evacuate the wells of liquid. Beads (1,000 beads/antigen/well) were suspended in Buffer A (PBS, 0.5% BSA, 0.05% Tween-20, 0.02% NaN_3_) and 50 µl bead master mix added to each well. Plates were washed two times with PBST, and 50 µL of the sample was added to each well and incubated with shaking at room temperature for 90 min. After 3 washes with PBST, detection antibodies corresponding to the sample type were added to wells and allowed to incubate for 45 min: biotinylated goat anti-rabbit IgG (H + L, 1:500, Zymed); biotinylated rat anti-mouse IgG (1:500, Invitrogen); or biotinylated mouse anti-human IgG (mixture of 1:500 × anti-hIgG and 1:625 anti-hIgG_4_, both Southern Biotech). Following incubation with detection antibodies, plates were washed three times, and beads were incubated with streptavidin–phycoerythrin (1:200 Invitrogen, Waltham, MA) for 30 min. Plates were washed three times and wells were incubated with Buffer A for 30 min under light shaking to remove any loosely bound antibodies. After an additional wash, samples were resuspended in 100 µl PBS and fluorescence data collected immediately on a MAGPIX with Bio-Plex Manager™ MP software with a target of 50 beads per region per well. Median fluorescence intensity (MFI) signal was generated for a minimum of 50 beads/region, and background MFI from wells incubated with Buffer B was subtracted from each sample to give a final value of MFI minus background (MFI-bg) for analysis.

### Immunofluorescence assays

*Plasmodium falciparum* NF54 thin smears were made from culture at the schizont stage, where parasitemia was determined to be 4%. Sera samples obtained from terminal bleeds of rabbits, diluted at 1:500 in PBS with 1% BSA, were used to evaluate antibody cross-reactivity against *P. falciparum* MSP1 via indirect immunofluorescence assay, as described previously [35]. Reactivity of immunized rabbits was compared to that of rabbit sera obtained prior to immunization with PvRMC-MSP1. A human hyperimmune pool of plasma with high reactivity to all *Plasmodium* MSP1 proteins was created from samples previously tested by serological assays. Immunofluorescence images were captured using a Zeiss confocal fluorescence microscope with Axiovision from Carl Zeiss MicroImaging GmbH (Thornwood, NY) software and presented at 100 × magnification.

### Genomic alignments and data analyses

All graphs were made in Microsoft Excel (Redmond, WA). Protein alignments were assessed with the Clustal Omega Multiple Sequence Alignment tool available from the European Bioinformatics Institute using the GenBank accession numbers corresponding to the species and strain used: XP_001352170.1 for *P. falciparum* 3D7, SCQ16291 for *P. ovale*, and SBS84075.1 for *P. malariae*. Alignment between a *P. vivax* SAL-I MSP-1 fragment (GenBank AAC37237.1) with the sequence of *P. vivax* Belem was also performed using the Clustal Omega Multiple Sequence Alignment tool. All sequences were compared to those reported for PvRMC-MSP1, which is based on the *P. vivax* Belem sequence, with accession number XP_001614842.1 [30].

## Results

### Sequence identity of PvRMC-MSP1 with other human *Plasmodium* species

The chimeric PvRMC-MSP1 protein has an estimated molecular mass of 31 kD and is schematically shown in Fig. 1a. All five promiscuous T cell epitopes included in PvRMC-MSP1 were derived from *P. vivax* Belem and selected for their ability to be broadly recognized by individuals with diverse HLA haplotypes. However, the previously determined T cell epitopes Pv19 and Pv53 to also function as B cell epitopes [31]. When the sequences of the five *P. vivax* MSP-1 promiscuous T cell epitopes included in the chimeric protein were compared to the sequences of the other *Plasmodium* species, it was observed that levels of amino acid identity ranging between 35 and 85% between PvRMC-MSP1 and the other human *Plasmodium* species (Fig. 1b). The epitope with the highest overall amino acid identity between *Plasmodium* species was PvT8 (average 82% across all species), while the lowest were PvT4 and PvT19 (both average 42% across all species). Considering all five T cell epitopes, *P. ovale* shows the highest identity with *P. vivax* (59% overall) followed by *P. malariae* (52%) and *P. falciparum* (50%). Among the MSP1_19_ fragments, *P. ovale* again showed the highest identity with PvRMC-MSP1 (51%), followed by *P. malariae* (50%) and *P. falciparum* (45%) (Fig. 1c).

In addition to assessing the sequence identity between *P. vivax* Belem, from which PvRMC-MSP1 is derived, alignment was carried out between the 1751 amino acid sequence available from GenBank for *P. vivax* Belem to that of a fragment of *P. vivax* Sal-I (GenBank AAC37237.1) due to the prominence of this isolate in Southeast Asia. Unfortunately, the published Sal-I fragment is only 101 amino acids in length, but comparison between the two sequences revealed only a single base-pair mutation from K to E between the Belem and Sal-I isolates at position 1709.

### Assessment of PvRMC-MSP1 capacity to elicit cross-reactive antibodies

**Fig. 2:**
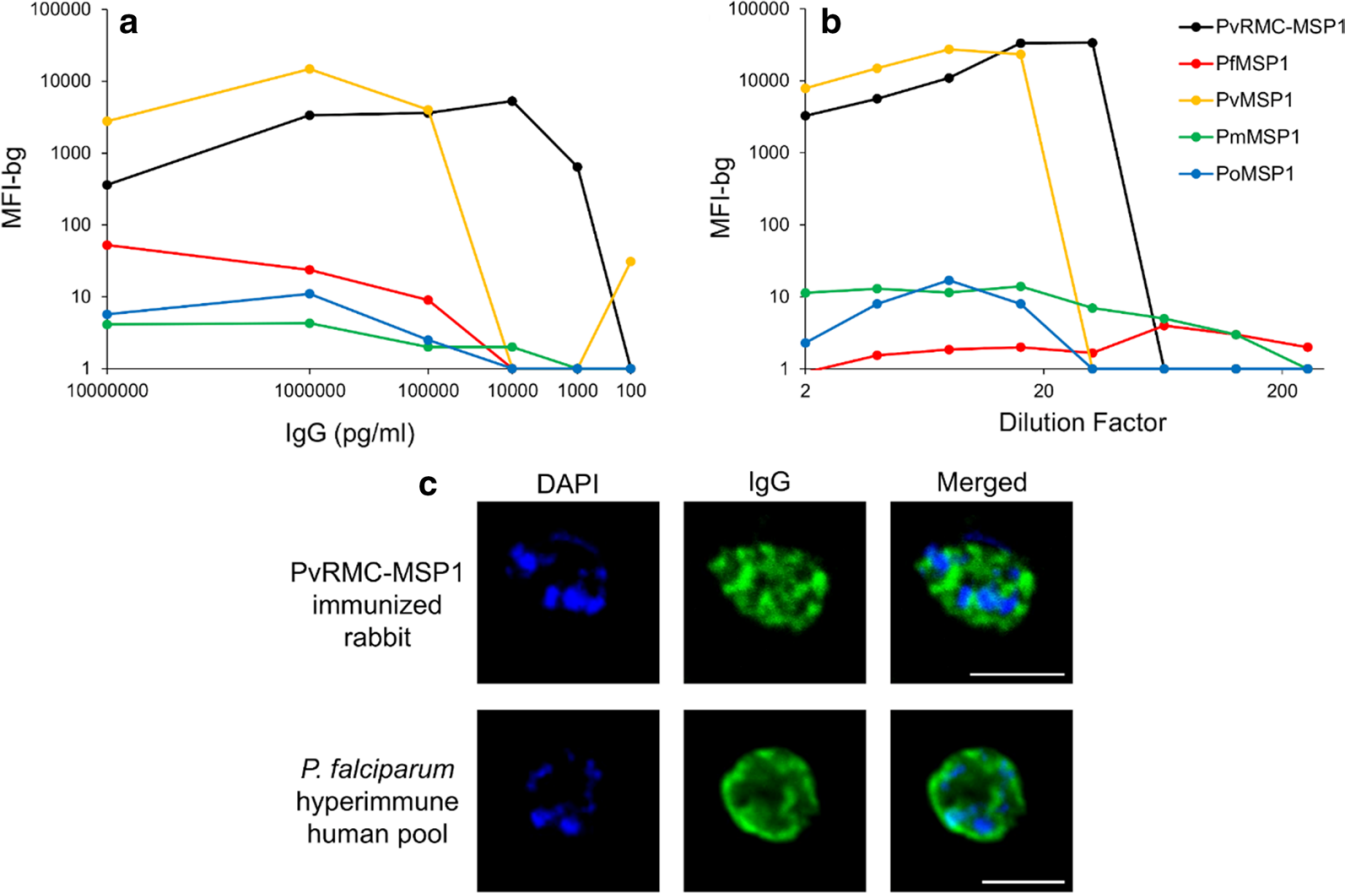
Capture of rabbit and murine anti-PvRMC-MSP1 antibodies with MSP1 proteins from the four *Plasmodium* species. **a** IgG assay signal for purified total IgG obtained from rabbits following four immunizations with the PvRMC-MSP1 protein. Median fluorescence intensity minus background (MFI-bg) assay signal is shown for each of the four MSP1 proteins derived from human *Plasmodium* species in comparison to the PvRMC-MSP1 used for immunization. **b** IgG assay signal for mouse sera obtained after three immunizations with PvRMC-MSP1 with MSP1 proteins from the four human *Plasmodium* species. **c** *Plasmodium falciparum* NF54-infected red blood cells smeared on glass slides were incubated with sera obtained from immunized rabbits (top), and plasma from individuals living in malaria*-*endemic regions (bottom). Panels show staining for DNA (DAPI, blue), IgG (AlexaFluor 488, green), and merge. All images are shown at 100 × magnification, and the scale bar indicates 5 µm

To determine if PvRMC-MSP1 could serve as a serological tool for assessing malaria exposure, sera or purified IgG derived from rabbits and mice previously immunized with PvRMC-MSP1 was used to measure the antibody binding capacity to the immunogen or the recombinant MSP1 proteins derived from the four human *Plasmodium* species. Purified total IgG from rabbits maintained a high reactivity to PvRMC-MSP1 before dropping off below 1000 pg/ml (Fig. 2a). The IgG assay reactivity to PvMSP1 also kept a high signal before dropping between 100,000 and 10,000 pg/mL. In addition to IgG binding to the proteins based on *P. vivax,* it was observed that a low-level signal at dilutions between 10^7^ and 10^4^ pg/ml for PfMSP1 and a very low assay signal for both PoMSP1 and PmMSP1. Assessment of sera derived from immunized mice resulted in a similar binding pattern, with the strongest signal elicited by PvRMC-MSP1, followed by *P. vivax* MSP1 (Fig. 2b). Very low assay signals were recorded for the immunized mouse sera against PfMSP1, PoMSP1, and PmMSP1, and did not appear to titrate out with further dilution. Immunofluorescence assays (IFAs) with slides fixed with parasitized red blood cells from *P. falciparum* NF54 culture showed IgG binding to *P. falciparum-*infected red blood cells from both PvRMC-MSP1-immunized rabbit sera as well as hyperimmune human sera (Fig. 2c), highlighting that IgG raised against the chimeric protein is capable of recognizing the native structure of *P. falciparum* MSP1.

### Binding of IgG from malaria-infected humans to PvRMC-MSP1 and MSP1_19_ proteins

**Fig. 3:**
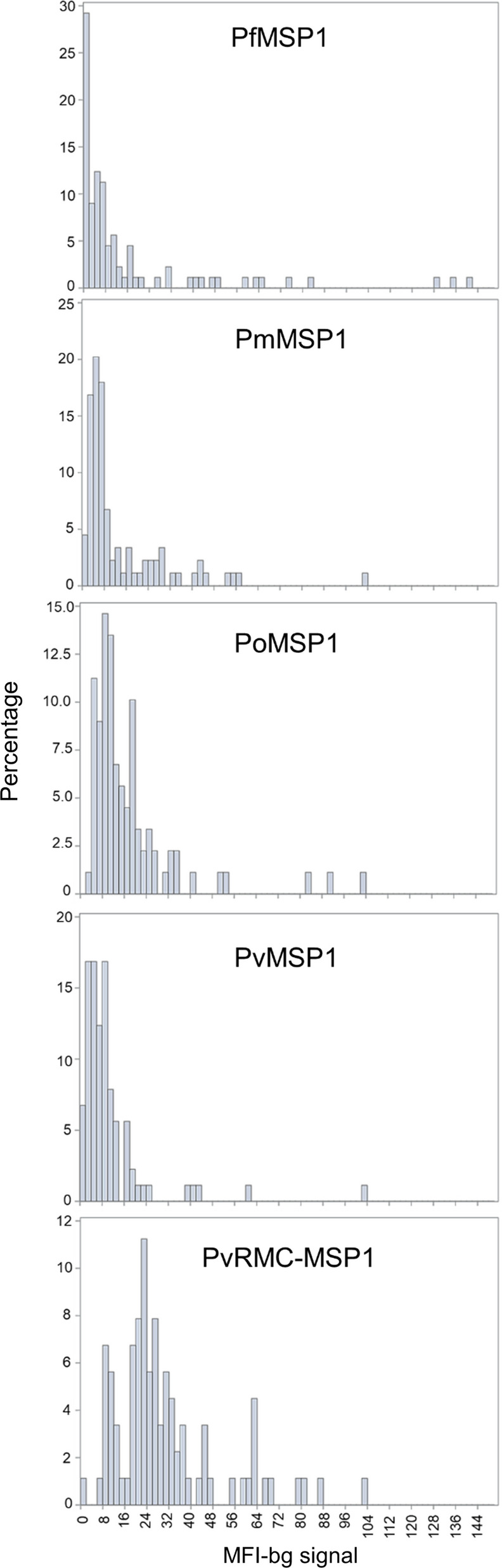
IgG assay signal for malaria naïve persons for PvRMC-MSP1, PfMSP1, PmMSP1, PoMSP1, and PvMSP1 antigens. Histograms display MFI-bg assay signal for a panel of 92 blood samples from persons never infected with malaria parasites. Note that x-axis is the same for all plots

Having demonstrated the ability of the chimeric protein-induced IgG to bind non-vivax antigens, the ability of IgG antibodies elicited during natural infection was investigated with human malaria parasites to bind the chimeric PvRMC-MSP. For these experiments, a panel of 236 plasma samples was used which were collected from travellers returning to the US with diagnosed malaria infections and a control panel of 92 malaria-naïve individuals. It was found the median fluorescence intensity minus background (MFI-bg) signals for naïve individuals to be consistently low in response to the chimeric PvRMC-MSP1 and all recombinant MSP1 proteins (Fig. 3).

**Fig. 4:**
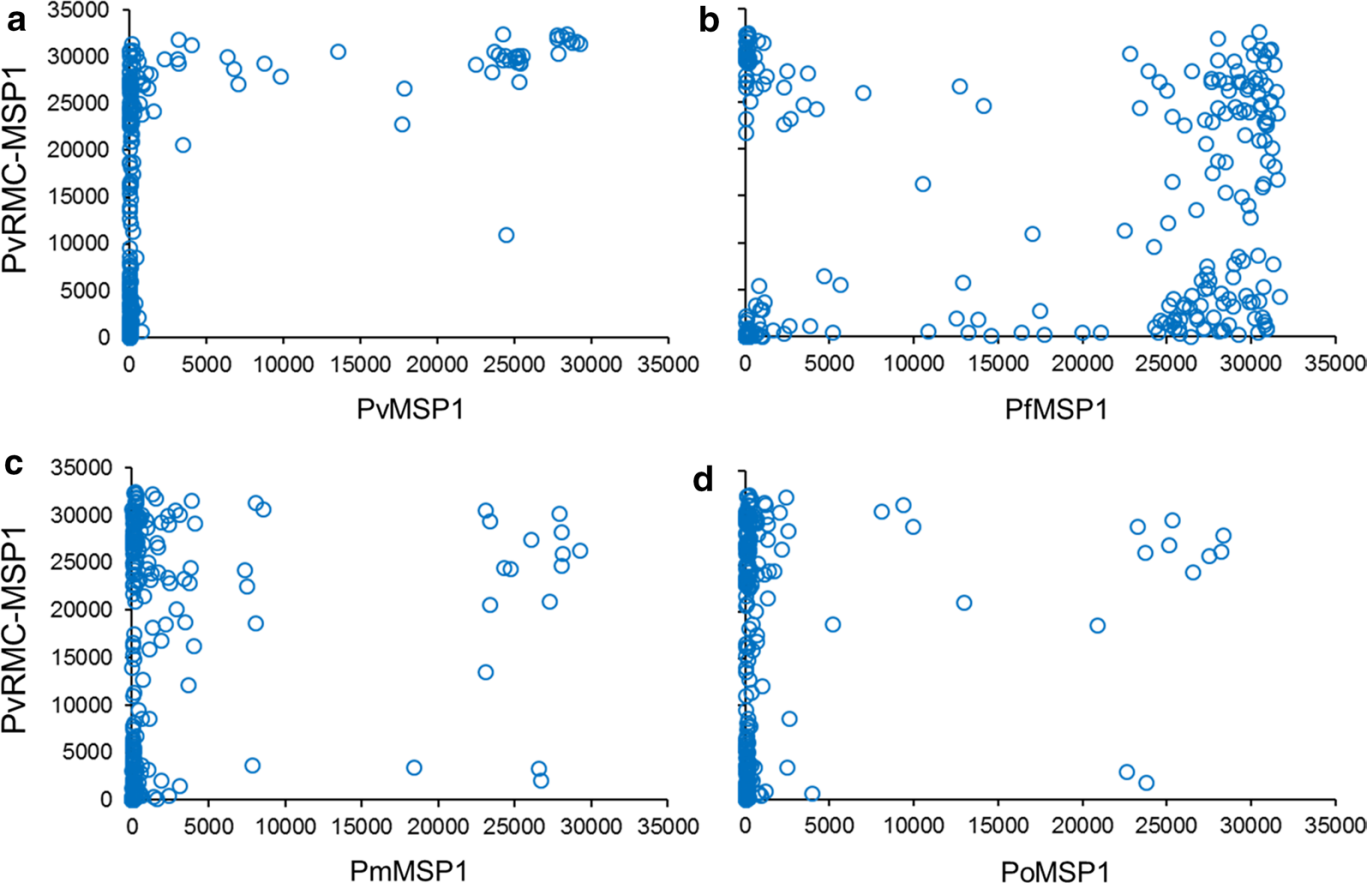
Comparison of MFI-bg assay signal for PvRMC-MSP1 and MSP1s recombinant proteins from the four human *Plasmodium* species for individuals with active malaria infection**.** Each point of the scatterplot displays an individual’s MFI-bg IgG response against PvRMC-MSP1 (y-axis) and the MFI-bg response from the same individual against the recombinant MSP1 19 kD proteins from one of the four human *Plasmodium* species (x-axis). Data was generated using plasma samples obtained from 236 returning US travellers with active malaria infection

As the goal was not to quantitate the level of response to PvRMC-MSP1, but rather determine if PvRMC-MSP1 could be used to capture antibodies from individuals naturally exposed to any of the four main *Plasmodium* species responsible for malaria in humans, assay signals were compared without strict positive or negative cutoff values for seropositivity. The ability of antibodies from malaria naturally-exposed individuals was assessed for binding to the same panel of recombinant MSP1 proteins representing the four human *Plasmodium* species and the chimeric PvRMC-MSP1 (Fig. 4). However, since the primary interest is in individuals that have high levels of antibodies capable of recognizing these proteins, a MFI-background threshold of 10,000 was selected in order to discuss “high responders” compared to “low responders”.

For the correlation of signal between PvRMC-MSP1 and PvMSP1, a dense cluster of 25 individuals, equivalent to 10.6% of the 236 traveller samples assayed, were double-positive individuals are observed (Fig. 4a). In addition, many individuals exhibit a strong signal to PvRMC-MSP1 (25, 10.6%) but a negligible signal for PvMSP1. As expected, no samples (0, 0.0%) provided a signal for PvMSP1 without also responding to PvRMC-MSP1 since the sequences included in the chimeric protein were derived from the PvMSP1 antigen.

Similarly, when the signal to PvRMC-MSP1 was compared to PfMSP1 for all 236 US traveller samples, four populations could be clearly observed with few samples in the middle range of assay signals (Fig. 4b). It was observed 145 individuals (61.4%) displayed only high signals for capture with the PfMSP1 antigen, 75 individuals (31.8%) positive for reactivity to both PfMSP1 and PvRMC-MSP1, and 115 individuals (48.7%) responding only to PvRMC -MSP1.

Comparison between PvRMC-MSP1 binding and either *P. malariae* or *P. ovale* MSP1 (Fig. 4c, d, respectively) produced similar trends where most individuals that responded to the PmMSP1 or PoMSP1 antigen also responded to PvRMC-MSP1 resulting in many more double-positive individuals than PmMSP1 or PoMSP1 single-positive individuals alone. For *P. malariae*, 15 individuals (6.4%) responded to the recombinant PmMSP1 protein alone, and 12 individuals (5.1%) that responded to both PvRMC-MSP1 and PmMSP1. When antibody capture was compared between PvRMC-MSP1 and PoMSP1, it was observed that 10 individuals (4.2%) that were high responders for both antigens, and 12 (5.1%) that responded to only PoMSP1. The number of individuals that responded only to PvRMC-MSP1 (115, 48.7%) remained the same for MFI-background signals obtained from binding to PfMSP1, PmMSP1, and PoMSP1.

**Fig. 5:**
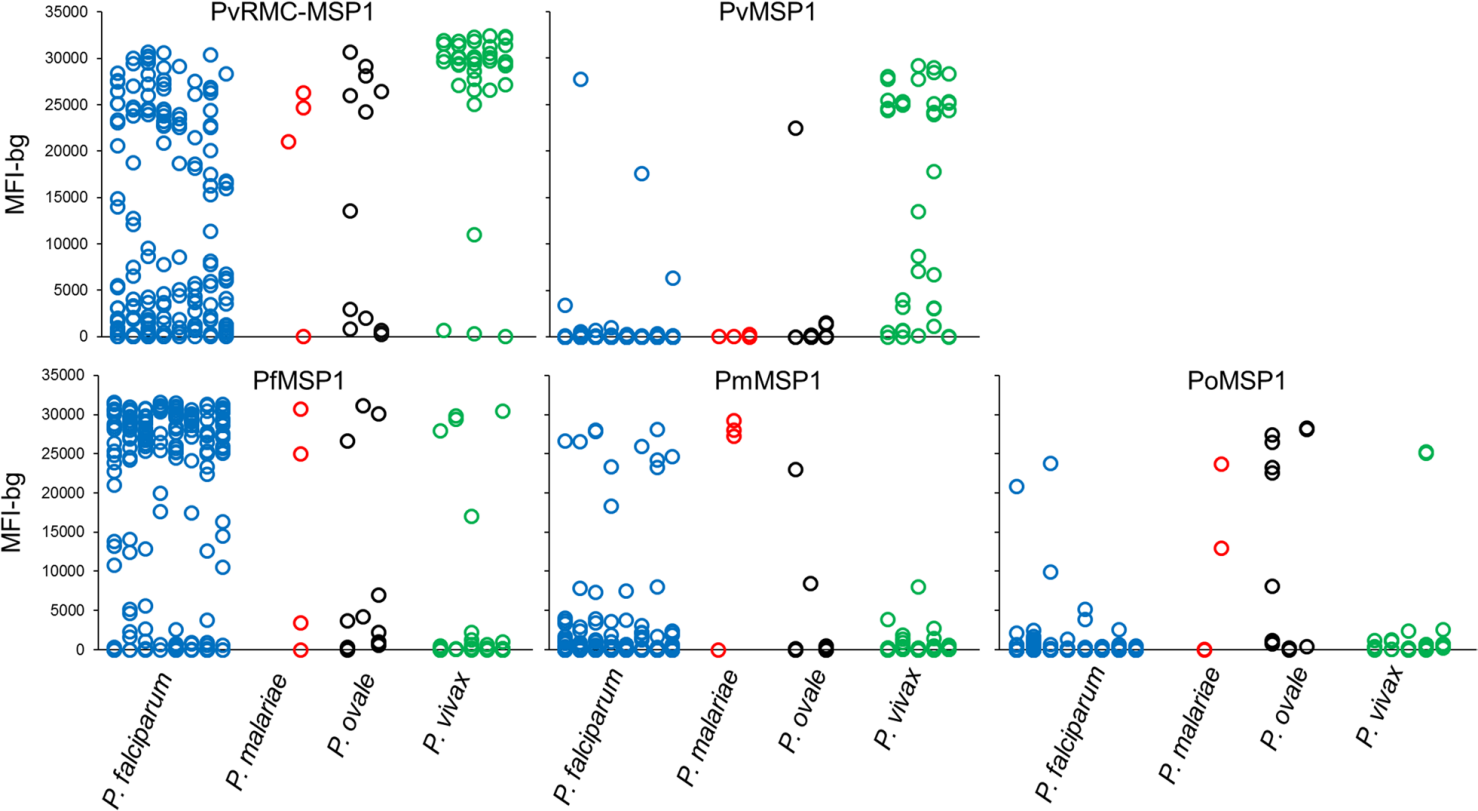
MFI-bg assay signal for PvRMC-MSP1 or *Plasmodium* MSP1 19 kD proteins grouped by active infection. Assay signal for IgG binding to a particular antigen is shown by each panel: PvRMC-MSP1 (top left), PvMSP1 (top right), PfMSP1 (bottom left), PmMSP1 (bottom middle), PoMSP1 (bottom right). Persons with active malaria infection categorized by infecting species: *P. falciparum* (n = 181, blue circles), *P. malariae* (n = 4, red circles), *P. ovale* (n = 13, black circles), *P. vivax* (n = 38, green circles)

To further understand how IgG produced by malaria infection was able to bind PvRMC-MSP1, plasma samples were categorized by species responsible for active infection. Of the 236 plasma samples from individuals with confirmed malaria infection, 181 were from *P. falciparum* infections, 4 were from *P. malariae*, 13 were from *P. ovale*, and 38 were from *P. vivax.* Regardless of the *Plasmodium* species responsible for infection, a large proportion of plasma from malaria-infected individuals provided a high assay signal to the PvRMC-MSP1 protein (Fig. 5), with only *P. falciparum-*infected individuals failing to have more than 50% of infected individuals with high responses to PvRMC-MSP1. Of the 181 *P. falciparum*-infected individuals, 70 individuals were seen with high responses to PvRMC-MSP1 (38.7% of 181 *P. falciparum* infections). For the 38 *P. vivax* infected individuals, 35 of 38 individuals (92.1%) had high MFI-background signal to PvRMC-MSP1. Similarly, 3 of 4 *P. malariae*-infected travellers had high responses to the chimeric protein (75.0%), and for the 13 *P. ovale-*infected individuals, 7 were classified as high responders to the PvRMC-MSP1 (53.4%).

When determining the assay signals grouped by infecting species for PvMSP1, it was found that for the 181 *P. falciparum* infected individuals binding to PvMSP1, there were only 2 high responders (1.1%). In stark contrast, IgG from *P. falciparum*-infected plasma binding to PvRMC-MSP1 gave a range of signals for many of the falciparum infections, with several of these approaching the maximum signal for the platform (MFI-bg signal of 32,000). It was observed that 70 *P. falciparum-*infected individuals had high responses to PvRMC-MSP1 (38.7% of the 181 *P. falciparum* infections).

**Fig. 6:**
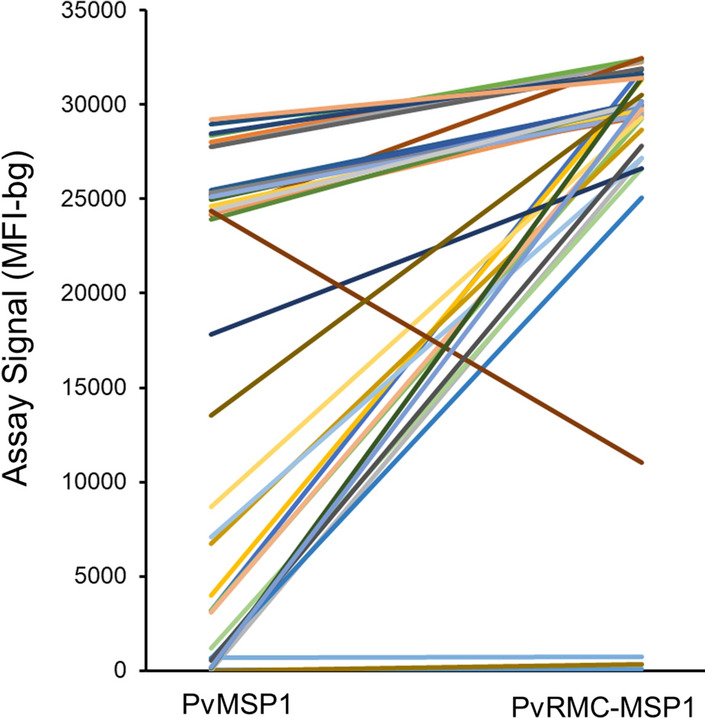
Recognition of recombinant PvMSP1 and PvRMC-MSP1 by individual *P. vivax* patients. Assay signals for individual patients are plotted as MFI minus background for both *P. vivax* MSP1 recombinant proteins tested. Each coloured line represents the change in signal between antigens for a single patient

When compared to the antibody binding between PvRMC-MSP1 and recombinant PvMSP1 using plasma from the 38 *P. vivax*-infected individuals, many of these samples neared the maximum signal intensity of the assay for PvRMC-MSP1, while a range of signals was observed for PvMSP1 alone. When the threshold set for high responders was applied, of 10,000 MFI-background signal, 35 of 38 *P. vivax-*infected individuals (92.1%) were high responders to PvRMC-MSP1 compared to the 22 out of 38 *P. vivax-*infected individuals (57.9%) that responded to PvMSP1 (Fig. 5). Upon closer evaluation, it was seen that the MFI-bg signals obtained from *P. vivax*-infected individuals binding to PvRMC-MSP1 were higher than that observed for binding to PvMSP1 in 34 out of 38 individuals (89.5%) (Fig. 6). Of the four *P. vivax*-infected individuals that did not show an increased signal for PvRMC-MSP1, a single sample produced decreased assay signal, and three samples produced low assay signals for both PvRMC-MSP1 and PvMSP1.

### Antibody binding to PvRMC-MSP1 from *P. falciparum-*infected individuals is not due to the presence of the PfCSP NANP repeat region alone

**Fig. 7:**
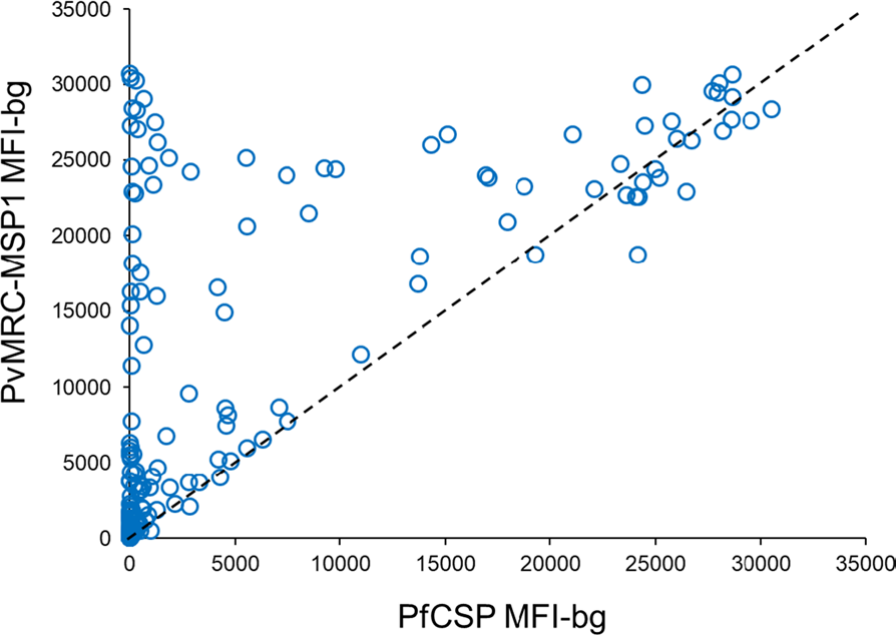
Cross-binding of anti-PfCSP IgG with PvRMC-MSP1. A scatterplot of the PfCSP signal in comparison with PvRMC-MSP1 for the 181 individuals with *P. falciparum* infection. The dashed reference line is shown as y = x for the assay signal

Due to the presence of six copies of the NANP amino acid sequence derived from the major repeat domain of *P. falciparum* CSP on the C-terminus of PvRMC-MSP1, it was investigated if the PvRMC-MSP1 binding signal observed from individuals with active *P. falciparum* malaria infection was due to IgG binding to the (NANP)_6_ alone. A scatterplot for the PfCSP and PvRMC-MSP1 assay signals for only the 181 individuals infected with *P. falciparum* showed that many samples were double-positive, responding to both of these antigens (Fig. 7). Additionally, some plasma samples showed a correlation of assay signals between the two antigens, tracking on a y = x reference line. However, some of these assay signals from *P. falciparum* infections were non-existent for PfCSP yet showed very high PvRMC-MSP1 IgG binding. No samples were IgG positive alone to PfCSP.

## Discussion

Numerous new tools have been developed for mass screening of populations to detect malaria exposure. In the field, these tools are typically designed to assay for some components of *Plasmodium* parasites to verify active infections. DNA and proteins produced during the parasite life cycle are attractive targets due to their high sensitivity and specificity in the detection of active infection [36, 37]. However, especially in areas of low malaria transmission, tools for detection of infection, including rapid diagnostic tests, microscopy, and PCR, can only confirm that infection prevalence is low in the studied population but do not offer data on the exposure history of survey participants. In contrast, the history of individual and population-level exposure can be determined by assessing host-produced antibodies and the window of time for finding a “malaria positive” positive is greatly increased [8, 25].

Serological studies offer numerous advantages over other measures of prevalence and transmission. Utilizing traditional diagnostics with passive surveillance of non-symptomatic persons in a region would underestimate the number of active cases and prevalence [38]. Microscopy requires skilled staff and cannot detect missing low-density or submicroscopic infections, nucleic acid-based assays are costly for large scale studies and only detect active infection [6, 8], and entomological inoculation rates are laborious to collect, have strong biases in heterogeneous environments [10] and can be difficult to extrapolate to the paediatric population who are most at risk of complications resulting from *Plasmodium* infection [11, 12].

For malaria serological studies, tailoring the sensitivity of the assay using a highly antigenic protein has previously been suggested as a method to improve serological testing and the estimates generated by this data [8]. A recent assessment of a multi-epitope chimeric protein for use as a serological marker in *P. falciparum* elimination settings in Southeast Asia demonstrated the potential of chimeric proteins in serological studies [28]. Another protein chimera based on the fusion of the *P. falciparum* MSP1 and MSP8 antigens has shown efficacy for the induction of growth-inhibitory antibodies in animal models [39, 40], but has been yet to be assayed against human plasma from naturally-exposed populations.

Based on data generated using these types of engineered antigens for malaria-based serological surveys, as well as our previous report on the high frequency of antibodies to PvRMC-MSP1 in individuals living in a malaria endemic area, it was sought to assess the ability of PvRMC-MSP1 to act as a pan-*Plasmodium* antigen target for serological surveys. Assessment of the conservation between PvRMC-MSP1 based on *P. vivax* Belem and the three other major *Plasmodium* species infecting humans revealed that for the extended 19 kD fragment of the MSP1 antigen, there is an approximately 50% conservation of the amino acid identity between the MSP1 orthologous regions and PvRMC-MSP1.

Previous publications on human antibody binding to the MSP1 19 kD antigen have shown that there is some conformational dependency and that anti-MSP1 antibodies tend to recognize epitopes that are conserved among variant sequences [41]. However, multiple motifs have been identified within the PfMSP1 19 kD protein which are available for antibody binding [41, 42], so sequence similarity of a single epitope would not necessarily dictate potential for cross-binding and IgG capture.

Sequence identity among the five promiscuous T cell epitopes present in PvRMC-MSP1 varied widely—from 35 to 85%, with the two epitopes that also function as B cell epitopes, PvT19 and PvT53, displaying amino acid identity ranging between 35 and 55%. This degree of conservation in the MSP1 and T cell epitopes provides a potential explanation for the binding of anti-PvRMC-MSP1 rabbit IgG to *P. falciparum* blood-stage schizonts and the ability of PvRMC-MSP1 to capture IgG from travellers infected with heterologous *Plasmodium* species. However, the sequence similarity or conformation of the 19 kD fragments alone would not provide a likely explanation for this finding, as human IgG among these antigens from different human *Plasmodium* species is largely species-specific [26].

After determining the level of homology between the MSP1 sequences of the major malaria parasites and PvRMC-MSP1, it was important to assess if this homology offered a functional significance that could allow anti-PvRMC-MSP1 antibodies to be used to capture different *Plasmodium* proteins. It was decided to test if anti-PvRMC-MSP1 IgG would be able to bind recombinant MSP1 proteins from the major human malaria species. As expected, anti-PvRMC-MSP1 antibodies had a high degree of binding with the PvRMC-MSP1 and the recombinant *P. vivax* MSP1 protein. Notably, it was observed that anti-PvRMC-MSP1 antibodies display cross-reactivity with the MSP1 proteins from *P. falciparum*, *P. ovale,* and *P. malariae*. While the overall degree of cross-binding with non-vivax malaria appears to be very low, the binding signal remains above the detection level even at high antibody dilutions for both rabbit and mouse induced anti-PvRMC-MSP1. Furthermore, it was able to be demonstrated that this cross-reactivity extends beyond recombinant proteins, as purified IgG from rabbits immunized with PvRMC-MSP1 bound to native *P. falciparum* MSP1 present in schizonts via IFA. The rabbit sera cross-binding to young parasite forms derived from culture displayed a similar binding pattern to that produced by *P. falciparum* hyperimmune human sera. This antibody binding pattern is also consistent with previous observations for IFAs using PvRMC-MSP1 immunized mice and *P. vivax* schizonts [30]. Combined, these data demonstrate that PvRMC-MSP1 can induce cross-reactive antibodies that are functional for the recognition of native malaria species.

Having confirmed that anti-PvRMC-MSP1 antibodies cross-react with non-vivax MSP1 proteins, the chimeric *P. vivax* MSP1 antigen was tested for its capacity to capture naturally induced IgG antibodies in plasma samples collected from US travellers with known active infection with one of the four major *Plasmodium* species that infect humans. While the species of malaria parasite for the current infection was known, the full medical histories for these individuals is unknown. Therefore, a limitation of this study was not knowing if a subset of individuals would have been exposed to the same or a different *Plasmodium* species previously and could display pan-species reactivity as a result of prior exposure. However, though the information was not available, as these are travellers returning to the US with malaria infection, it could be reasonably assumed that this is the first malaria exposure for many. High IgG titers are typically seen with active infection [24], so this set of plasma from infected persons provided species-induced IgG for which to assess cross binding.

When comparing the IgG capture signal between PvRMC-MSP1 and the four recombinant *Plasmodium* proteins using plasma from malaria-infected returning US travellers, irrespective of infective species, it was consistently observed a subgroup that was able to strongly recognize both PvRMC-MSP1 and the recombinant protein. However, while IgG capture between PvRMC-MSP1 and PmMSP1 and PoMSP1 was seen, due to the limited number of *P. ovale* (n = 13) and *P. malariae* (n = 4) samples included in this study, further assessment using a larger *P. ovale* and *P. malariae* exposed sample sets is required. This broad IgG binding capacity may be due to the selection of promiscuous T cell epitopes within the PvRMC-MSP1 protein, which included epitopes based on their predicted ability to bind multiple human MHC class II molecules. An assessment of synthetic peptides from *P. vivax* MSP1 preceding the development of PvRMC-MSP1 showed a high degree of variability in the ability of peptides to bind HLA class II molecules and to be recognized by individuals living in endemic areas of malaria [31].

Furthermore, data from the initial characterization of PvRMC-MSP1 demonstrated that 50.4% of individuals living in Brazil had antibodies able to recognize the PvRMC-MSP1 with no indication of genetic restriction based on HLA-DRB1 and HLA-DQB1* alleles [30].

During the assessment of plasma from travellers based on the cause of active infection, we observed that the majority of *P. vivax-*infected individuals carrying IgG antibodies had a higher degree of binding to PvRMC-MSP1 when compared to the recombinant PvMSP1 protein alone. Though these antigens were coupled to the microbeads at the same concentration, the assay signal of PvRMC-MSP1 versus PvMSP1 was increased for 89.5% (34/38) of *P. vivax-*infected plasma, with some samples showing an increase of over an order of magnitude. It is possible that the reorganization of the broadly recognized epitopes from PvMSP1 into the chimeric PvRMC-MSP1 protein, and the removal of sequences of low antigenic value, made the broadly recognized epitopes more accessible to antibody binding, thereby increasing assay signal. It has previously been reported circular dichroism studies of PvRMC-MSP1 [30], suggesting that the carboxyl-terminal region in PvRMC-MSP1 is in the same conformation as reported for native PvMSP1. Therefore, it does not appear the conformational changes to be the cause of increased binding to PvRMC-MSP1 over recombinant PvMSP1.

The increased binding observed for PvRMC-MSP1 over recombinant PvMSP1 may aid in the development of improved serological assays for use in *P. vivax* endemic regions approaching elimination. Population-based studies in these regions require highly sensitive assays as a decreasing infection burden leads to increases in the frequency of asymptomatic cases and lack of treatment-seeking behaviour, making detection of cases more challenging. Many regions of South and Central America are currently within or approaching the elimination phase of malaria control, and *P. vivax* is the predominant residual species in some of these areas [43]. Having a more sensitive serological tool for detection of *P. vivax* exposure would be able to assist the Americas and other global settings in mapping out regions and populations where malaria still resides. Furthermore, in countries within the Horn of Africa, *P. falciparum* and *P. vivax* both circulate, so serological studies based on PvRMC-MSP1 should be using in combination with recombinant PfMSP1 and PvMSP1 to obtain species specific reactivity in addition to the pan-*Plasmodium* signal that could result from use of PvRMC-MSP1 alone.

Designed antigens with intentional broad antibody binding capacity could provide a valuable tool for use in serological assessment studies when compared to whole recombinant antigens alone, which are intended to be true to the genome-encoded antigen. This current study benefited from the use of the bead-based multiplex immunoassay, which allows simultaneous data collection of IgG presence and levels against multiple antigens [20]. Including both broad-reacting, as well as *Plasmodium* species-specific, antigen-coated beads in an assay panel could provide a very nuanced view of individual and population-level exposure histories as well as provide an extensive IgG profile and detailed seroestimates [44, 45]. The population of US resident travellers returning with malaria infection would be more biased toward those with nascent exposure, but a limitation to this study is the lack of information on the number of previous episodes, or what *Plasmodium* species persons would have been previously exposed to in their lives. This possibility of previous lifetime exposure provides a reasonable explanation for the finding of IgG antibodies capable of binding the MSP1 proteins from the other, non-infecting, *Plasmodium* species.

Beyond known active *Plasmodium* infections, future studies will work to assess antibody binding to PvRMC-MSP1, and other chimeric antigens, to naturally exposed human populations in different regions of the world with different transmission intensities and co-endemic patterns. Access was not available to any *Plasmodium knowlesi*-infected US travellers during the sample collection period. Due to the increasing importance of this *Plasmodium* species in Southeast Asia, future studies should be conducted to assess the ability of this broadly recognized PvRMC-MSP1 protein to capture antibodies generated as the result of *P. knowlesi* exposure. Furthermore, the dynamics of the anti-*Plasmodium* antibody response, and particularly anti-MSP1 antibodies, is not well established. It remains to be evaluated how quickly these antibody responses form in naïve individuals and how durable these antibody responses are, with consideration given to age and transmission intensity.

## Conclusions

Overall, this study offers insights into the field of multi-species malaria serology and the potential for improving multiplex serological assays for estimating the burden of malaria on human populations. As malaria surveillance is typically focused on a predominant one (or two) *Plasmodium* species, improved methods for identifying and monitoring foci of active transmission and multi-species endemicity are needed. Current multiplex serology approaches have been generally limited to recombinant proteins that attempt to mirror in sequence and structure the native antigen produced by the malaria parasite.

Here the reactivity of antibodies derived from immunized animals and naturally infected US travellers to recognize a chimeric *Plasmodium vivax* recombinant protein is described. Regardless of infecting *Plasmodium* species, a majority of plasma IgG antibodies provided a high assay signal to the PvRMC-MSP1 chimeric protein. Assay signals were also increased for PvRMC-MSP1 compared to the recombinant *P. vivax* MSP1. These findings support the further study of other engineered antigens as a means of improving existing serological tools. As presented here in the multiplex platform, chimeric or engineered antigens can be assayed simultaneously with other recombinant antigens for the detection of antibodies to create a very clear picture of active or historical malaria exposure for an individual.

## Data Availability

Data and materials used during the current study are available from the corresponding author upon reasonable request.

## Abbreviations

AMA1: Apical membrane antigen 1
BSA: Bovine serum albumin
CSP: Circumsporozoite protein
EIR: Entomological inoculation rates
HLA: Human leukocyte antigens
IFA: Immunofluorescence assay
IgG: Immunoglobulin
MFI: Median fluorescence intensity
MHC: Major histocompatibility complex
MSP1: Merozoite surface protein 1
MSP1 19 kD or MSP1_19_: MSP1 19 kilodalton fragment
MSP1_33_: MSP1 33 kilodalton fragment
PBS: Phosphate buffered saline
PBST: Phosphate buffered saline with Tween20
PfCSP: *Plasmodium falciparum* Circumsporozoite protein
PfMSP1: *Plasmodium falciparum* MSP1
PmMSP1: *Plasmodium malariae* MSP1
PoMSP1: *Plasmodium ovale* MSP1
PvMSP1: *Plasmodium vivax* MSP1
PvRMC-MSP1: *P. vivax* Recombinant Modular Chimera based on Merozoite Surface Protein 1
RT: Room temperature
